# From the Operating Room to the Managerial Table: Healthcare Management Lessons and Transformative Leadership of an Orthopedic Surgeon at a Public Tertiary Hospital in South-West Nigeria

**DOI:** 10.7759/cureus.99717

**Published:** 2025-12-20

**Authors:** Aminat E Akanbi, Olanrewaju S Jimoh, Solihat U Yesufu

**Affiliations:** 1 Department of Family Medicine/General Practice, Federal Medical Centre, Epe, NGA; 2 Department of Public Health, University of Ilorin College of Health Sciences, Ilorin, NGA; 3 Department of Obstetrics and Gynaecology, Federal Medical Centre, Abeokuta, NGA; 4 Department of Medicine, University of Ilorin Teaching Hospital, Ilorin, NGA

**Keywords:** clinical leadership, digital health, electronic medical records (emr), healthcare governance, health systems management, hospital reform, nigeria, quality improvement, universal health coverage (uhc), workforce development

## Abstract

This qualitative editorial draws on a single-case, semi-structured consented interview conducted in July 2025, triangulated with publicly available institutional reports and data from 2017 to 2025, to examine leadership-driven hospital improvement.

It presents lessons from an orthopedic surgeon who transformed a public tertiary healthcare facility in South-Western Nigeria into one of the nation’s most digitally advanced and operationally efficient public health institutions. The editorial highlights the five-pillar strategy that guided system-wide reforms, which were later adopted by over 70 institutions nationwide. His leadership championed clinician-informed governance, workforce stability, trust, and public accountability. By discouraging unsafe care practices and integrating clinical insight into administrative leadership, this model demonstrates how specialty-trained clinicians can drive institutional change, inform policy, and promote health equity in low and middle-income countries.

## Editorial

In many low and middle-income countries (LMICs), hospital systems face persistent challenges, such as outdated infrastructure, fragmented recordkeeping, unreliable digital tools, workforce instability, and limited financial resources. Although international policy initiatives and donor programs have aimed at addressing these gaps, lasting changes are often driven by internal leadership, especially when clinicians who are familiar with the patient needs also engage in administrative reform. When such leadership emerges from specialized disciplines like surgical specialties, where cases and functional outcomes directly intersect with system design, it creates opportunities for pragmatic and impactful change.

Problem context

Before 2017, the Federal Medical Centre (FMC) Ebute-Metta faced typical challenges of LMIC tertiary hospitals, including manual workflow, fragmented record systems, equipment shortages, and unstable labor relations. These inefficiencies resulted in prolonged patient wait times and limited service capacity.

Aim

This article examines the leadership-driven transformation of the FMC Ebute-Metta, focusing on the role of Dr. Adedamola Dada, an orthopedic surgeon and administrator, and explores quality-improvement principles that can be replicated in other resource-constrained hospitals.

Dr. Adedamola Dada served as the Medical Director (MD) and Chief Executive Officer (CEO) of FMC Ebute-Metta in Lagos from 2017 to 2025, leading its transformation into one of Nigeria’s most digitally advanced and operationally efficient public facilities. Media reports highlighted Dr. Adedamola Dada's professional reputation and leadership style, noting their contribution to improvement in hospital operations [1].

“In 2017, when I assumed the role, the hospital had a whole systemic issue as there were multiple challenges, not limited to clinical units, and quite a number of them still exist in similar facilities today.” Dr. Dada explained during our interview, “We simply did an environmental scan to know where we were and plan where we would want to be. This led to the development of a strategic plan, which was based on what we could do then, what and where we wanted to be, the kind and the quality of services we wanted to render to Nigerians.”

This editorial explores the public health and health systems lessons embedded in Dr. Dada’s leadership experience. While his reforms spanned every department, his clinical training subtly shaped aspects of infrastructure, workflow, and healthcare access planning. The insights presented here underscore how clinician-based hospital leadership can contribute to systems innovation, sustainable institutional reform, and promote equity in healthcare access in resource-limited settings.

Method of source of evidence

This editorial employs a qualitative, narrative single-case study approach to explore leadership strategies and institutional performance at FMC Ebute-Metta. Data were obtained through a recorded, consented semi-structured interview conducted in July 2025 with Dr. Adedamola Dada, complemented by secondary reports documenting hospital performance from 2017 to 2025. Transcripts were manually reviewed and thematically analyzed, with emergent themes coded into domains aligned with the hospital's five-pillar strategic framework. Statements corroborated by secondary institutional reports were retained to strengthen validity and reduce interpretation bias. No patient-identifiable data were used, and ethics approval was not required.

Strategic transformation of FMC Ebute-Metta

When Dr. Adedamola Dada assumed leadership in 2017, the hospital faced systemic challenges common to public tertiary centers in Nigeria, including manual operations, outdated infrastructure, workforce instability, limited financing, and inefficient service delivery. Leveraging his clinical experience and administrative vision, Dr. Dada initiated a reform strategy grounded in what he described as “five key pillars of the strategic plan,” namely, human resources development, institutional stewardship, infrastructure expansion, financial modernization, and digital technology [2].

In the area of human resources development, the strategy's foundation was centered on staff capacity building. “We needed to re-orientate our workers, empower, motivate, train, and provide them with good leadership,” he explained. This pillar focused on training, leadership growth, and enhancing the well-being and work capacity of both clinical teams and administrative staff. This is evident from the sponsorship of clinical and non-clinical staff members for local and international conferences.

On the aspect of institutional stewardship, Dr. Dada emphasized the importance of ethical governance and accountability in leadership. “There was a roaring need to improve the quality of care and services that we were rendering to Nigerians,” he explained. He restructured the management approach to encourage transparency, teamwork, and shared responsibility, noting that institutional growth required members who understood that they were responsible for the system’s well-being.

Hospital infrastructure was substantially upgraded during his tenure. The health facility experienced substantial infrastructural upgrades across critical domains. In addition, the number of specialties, consultants, and training departments grew considerably, reflecting improved workforce capacity and institutional readiness to meet diverse population health needs. “There was a need for us to expand our services, improve their quality, renew our equipment, and refurbish our infrastructure,” Dr. Dada said. The summary of these infrastructure upgrades is presented in Table 1 [3].

**Table 1:** Summary of the infrastructure upgrades as of 2025. Adapted from *The* *Rebirth* *of* *Federal* *Medical* *Centre* *Ebute-Metta,* *2017–2025* [3]. Percentages calculated by the authors for context and have not been independently verified. LINAC: linear accelerator.

Equipment	2017 (N)	2017 (%)	2025 (N)	2025 (%)
Beds	75	55.1	420	20.7
Theater suites	2	1.5	12	0.6
ICU beds	0	0.0	16	0.8
Ambulances	1	0.7	11	0.5
Oxygen plant	1	0.7	2	0.1
Number of cylinders produced	12	8.8	120	5.9
CT scan	0	0.0	3	0.1
X-rays	1	0.7	4	0.2
Patient monitors	1	0.7	300	14.8
Ventilators	2	1.5	23	1.1
LINAC accelerator	0	0.0	1	0.0
Ultrasound	1	0.7	10	0.5
Mammography machine	0	0.0	2	0.1
Dialysis machines	5	3.7	13	0.6
Incubators	2	1.5	15	0.7
Solar inverters	0 sites	0.0	22 sites	1.1
Electronic medical record (EMR) infrastructure	0	0.0	926	45.6
Clinical training center	0	0.0	1	0.0
Training departments	2	1.5	10	0.5
Number of specialties	14	10.3	41	2.0
Consultants	17	12.5	78	3.8
Total	136	100.0	2030	100.0

When speaking of financial modernization, he emphasized the importance of updating, modernizing, and automating the hospital’s financial systems to improve transparency, eliminate leaks, and foster collaboration, stating that stakeholders are more willing to offer support when financial accountability is assured. In addition, he was able to create a partnership with organizations that were willing to support the hospital because he believes transparency and collaboration go hand-in-hand [3,4].

Regarding digital technology, the most visible and groundbreaking reform was the shift to an entirely paperless hospital system, integrating electronic medical records (EMR), laboratory information systems (LIS), and digital billing, as shown in Table 1. “The last component was the use of technology to enhance the quality of care,” he explained. “This approach allowed us to become the very first public institution in the country to attain paperless clinical operations.” The success of this digital ecosystem led over 70 public hospitals across Nigeria to adopt FMC’s EMR model [1,5].

Reflecting on the overall plan, Dr. Dada explained, “Virtually, all the pillars improved, not just the clinical-based care, but the entire care in the hospital. They had a positive impact on the quality of care provided to our patients. We became known as a place where you can certainly come to, be properly taken care of by our workers and be left with fewer reasons to complain.”

This strategic model positioned FMC Ebute-Metta as a national reference point for clinician-led institutional reform, demonstrating how aligned leadership and organizational vision can drive measurable system improvement. Dr. Dada’s background subtly informed both infrastructure design and specific systematic prioritization, demonstrating how specialty-informed leadership can drive meaningful reform in public health systems.

Baseline Interpretation

The 2017 figures reflect a low-capacity tertiary hospital with 75 beds, only two operating theaters, no intensive care unit (ICU) capacity, one ambulance, no X-ray machine, and no digital infrastructure. Following implementation of the five-pillar reform strategy, the 2025 data show progressive expansion of 420 beds, 12 theaters, 16 ICU beds, 11 ambulances, three CT scanners, full EMR deployment (926 terminals), and an increase in consultants from 17 to 78. Total equipment count rose from 103 to 1901. These contrasts quantify the magnitude of improvement and demonstrate the scale of pre-intervention constraints more clearly than a qualitative description alone.

Service Delivery and Patient Impact

Beyond infrastructure expansion, the reforms translated into measurable improvements in service delivery. EMR adoption eliminated clerical errors, improved turnaround time for laboratory and radiology results, and reduced documentation delays. Staff retraining and clear standard operating procedures (SOPs) lowered conflict and improved communication across patient touchpoints. Word-of-mouth referrals increased significantly as more patients reported satisfactory care experiences. New patient intake rose to approximately 5000-6000 new registrations per month, in addition to tens of thousands of returning patients. Increased theater capacity, ICU beds, and equipment availability reduced waiting time for procedures and improved continuity of care. These outcomes indicate that reforms directly strengthened efficiency, patient flow, and perceived quality of services.

Measured Outcomes

The transformation produced quantifiable improvements in institutional capacity; equipment inventory increased from 136 to 2030 units, digital tools expanded from zero to full EMR coverage across clinical units, and service specialties grew from 14 to 41. Monthly new patient intake rose to over 5000, reflecting increased public confidence and service efficiency. Importantly, waiting time for care was reduced, and feedback trends indicated higher patient satisfaction. Work disruption through labor strikes dropped to zero over an eight-year period, signaling both workforce trust and strong reform ownership.

Observed Challenges

Initial workflow disruption occurred during EMR adoption and infrastructure upgrades. Procurement pacing, user training, and adjustment to new digital processes required phased adaptation. However, sustained communication, continuous skill-building, and trust-based leadership helped mitigate resistance, allowing the reforms to stabilize and scale successfully.

From operating room to the managerial table: orthopedic surgery as a lens for health reform

In the architecture of LMIC health systems, trauma and orthopedic surgeons often occupy a critical yet often overlooked role. They are not simply clinical responders; they observed direct consequences of systemic failure, including untreated cases, preventable deformities, and irreversible complications. Dr. Adedamola Dada’s journey from trauma surgeon to reform-driven hospital leader exemplifies this intersection between clinical proximity and structural responsibility [6].

“Despite our enormous administrative work, we managed to fulfil some of our clinical responsibilities,” he stated. “This also allowed us to assess the system. By using the system, we could identify what was working well and what was not.”

Years of surgical practice had exposed him to the consequences of weak referral chains, misdiagnosis, and culturally embedded but unsafe practices, raising significant ethical concerns. As Medical Director of FMC, Ebute-Metta, Dr. Dada applied the urgency, accountability, and systems thinking of a trauma surgeon into the world of hospital governance. His orthopedic lens made him acutely aware of how delays, misinformation, and poor access compound over time, particularly in relation to traditional bone setting (TBS).

“Several scholars have considered the possibility of training traditional bone setters and incorporating them into the primary healthcare,” he said. “However, despite the evidence presented, I’m not convinced that it is the right approach. My reason is that they essentially appear to be impervious to corrections. It’s a bit of a problem in the healthcare system and is also evident in most areas of medicine, such as traditional birth delivery.”

Dr. Dada's perspective reflects his professional experience as narrated in this editorial. He warned against normalizing systems that fail to guarantee patient safety or continuity of care, given the significant morbidity and mortality risks. In addition, he proposed the need to establish structured strategies that either discourage or guide individuals away from seeking care pathways that risk exacerbating, rather than improving, their health conditions.

His position goes beyond TBS. He views much of the disease burden of the body systems in LMICs as cumulative results of policy neglect, delayed care, and system inertia. In this way, Dr. Dada offers a sharp redefinition of what it means to be a surgeon in LMICs. Rather than retreat into clinical silos, he encourages speciality clinicians to view themselves as architects of system change.

Mechanism of Change

His surgical background served not only as clinical expertise but also guided operational decision-making. Exposure to delayed referrals, poorly-managed fractures, surgical complications, and unsafe TBS practices sharpened priorities around EMR adoption, standardized triage, workforce accountability, and patient pathway protection. Clinical challenges informed priorities for system improvement, demonstrating how surgeon-leaders can translate micro-level experience into macro-level governance.

Effective leadership systems built on stability and the architecture of trust

*Systems* *Leadership* *and* *Workforce* *Harmony*

One of the most striking features of Dr. Adedamola Dada’s tenure at FMC Ebute-Metta was the absence of labor unrest or industrial strikes across eight years. In Nigeria’s public-sector healthcare, where strikes are common, this rare outcome reflected a strategic approach to inclusive leadership, workforce accountability, and communication [7].

“There was no magic to it. It was just the sincerity of leadership,” he stated. “During our leadership, we engaged our staff, were open to them, and avoided situations that would betray their trust.”

Dr. Dada and his management team established structured grievance mechanisms and maintained open, honest communication with staff. Rather than imposing reforms from the top, his leadership was transparent and involved departments in shaping the strategic vision. The staff members were empowered not only as implementers but as stakeholders, with space to question, recommend, and innovate. This inclusive model is extended to capacity building and career progression. In return, key departments remained stable, functional, and committed to shared goals.

*The* *Intangible* *Architecture* *of* *Trust*

Beyond structures and policies, his success was anchored in something less measurable but equally powerful, institutional trust. He fostered an environment where staff felt seen, heard, and safe. Reforms like digitization, EMR adoption, and infrastructure expansion were not met with resistance because the people they affected had already bought into the leadership itself.

This relational credibility came from consistency, accessibility, and clarity of intent. Team members across cadres saw in him a leader who acted not out of power, but out of purpose. His influence was driven less by charisma and more by moral consistency - a leadership style rooted in ethical public service.

“The workers saw that we were acting in their best interests, understood that they owned the institution and its process, and embraced a sense of ownership,” he noted during the interview. “If they requested resources needed for their jobs, they were certain the management would respond positively.”

This form of human-centered leadership created psychological safety, a necessary but often overlooked condition for sustainable institutional reform. In systems where workers fear retaliation or neglect, even the best-designed policies falter. At FMC, reforms thrived because the culture supported innovation, rather than mere compliance.

Dr. Dada’s story is a reminder that health system transformation is not just about infrastructure or digital tools. It is about the emotional foundation of institutional culture - the trust, legitimacy, and shared values that hold systems together when resources are scarce, and change is challenging.

Policy, access, and public health integration: Why health insurance reform is the missing foundation of systemic equity

Despite growing attention to digital health, hospital infrastructure, and workforce investment across LMICs, one foundational gap persists, which is inadequate financial protection at the point of care. In Nigeria, this gap is particularly acute, where only 3% to 5% of the population is covered by any form of health insurance. For Dr. Dada, this is more than a financing issue; it is the core policy failure that limits access, distorts care-seeking patterns, and reduces the visibility and utilization of healthcare services, including trauma and orthopedic services.

“Nobody should pay for healthcare at the point of need,” he explained during our interview. “There is nobody who keeps money for sickness in the bank.”

His call to action is clear: without a functioning, inclusive national health insurance system, all other reforms, whether in trauma, orthopedic, or general medicine, are building on nothing. The absence of coverage not only delays care but also redirects patients to informal or unregulated spaces like churches, mosques, and traditional homes, not because of preferences but because of affordability.

From his dual perspective as a surgeon and hospital administrator, Dr. Dada connects this financing gap to broader dysfunction: tertiary hospitals are overloaded with minor cases; primary care is underutilized or bypassed; non-emergency conditions that progress into costly, complex emergencies; and there is systemic neglect of non-communicable and musculoskeletal conditions.

“The most profound impact on our healthcare system would occur when our health insurance is functioning well,” he emphasized.

This vision aligns with the principles of the World Health Organization (WHO) on Universal Health Coverage (UHC), which stress that countries must expand both population coverage and service access while protecting patients from catastrophic health spending [8]. Dr. Dada’s experience shows that hospitals can innovate from within, but lasting equity and integration must be anchored in national financing reform.

An equally critical pillar of this vision is data integrity. He emphasized that without clean, stratified, and accessible population data, health planners cannot design a tiered, inclusive insurance model. Efforts to harmonize national identity tools (e.g., bank verification number, national identity number, and others) should be leveraged not just for security or voter rolls, but as instruments for population stratification and insurance policy planning.

“In law, they say you can’t build something on nothing,” he reflected. “We must get the foundation right.”

By focusing public energy and political will on health insurance and population data reform, Nigeria has the potential to reverse the fragmentation that currently marginalizes surgical and medical care from the national conversation. Dr. Dada’s policy view is not theoretical; it is shaped by eight years of leadership in a hospital that had to function despite this vacuum. His call is both practical and urgent [9,10].

Generalizability

Institutional reforms demonstrated feasibility - digital adoption, EMR integration, workforce growth - yet scalability requires national insurance expansion, integrated referral funding, and population data systems to enable risk pooling and ensure equitable access. These lessons are transferable to other Nigerian federal hospitals and comparable LMIC settings with similar financing gaps.

From vision to structure: planning as the lifeline of public health leadership

For many clinicians in LMICs, the pathway from bedside care to systemic leadership can feel unclear, obscured by bureaucracy, undermined by poor infrastructure, or burdened by burnout. But for Dr. Dada, the key to meaningful transformation was not charisma nor political leverage but structured planning.

“There is no secret and there is no magic,” he told us. “If you have a problem, you need to dissect the problem… design a plan that can resolve it.”

His response was not theoretical. It was the same structured approach that underpinned one of Nigeria’s most notable federal hospital transformations. During his eight-year tenure as the MD and CEO of FMC Ebute-Metta, Dr. Dada implemented sweeping reforms, all rooted in a deliberate and outcome-driven planning process.

*From* *Diagnosis* *to* *Design:* *Applying* *Clinical* *Thinking* *to* *Systems*

One of Dr. Dada’s most impactful insights is the idea that system leadership is not fundamentally different from clinical problem solving. Just as a surgeon diagnoses before intervening, he argues that health systems must be approached with the same discipline: observe, map, plan, act, and measure.

This is echoed in his insistence that institutional decisions must avoid ad hoc reactions and improvisation. Instead, he advocates for data-informed environmental scans, clear objectives, and measurable metrics, principles that echo foundational models of public health management and quality improvement.

“If you fail to plan,” he cautioned, “then you are planning to fail.”

*The* *FMC* *Model:* *From* *Vision* *to* *Implementation*

The planning framework he describes was not abstract. It served as the launch pad for the FMC’s five-pillar transformation strategy.

His leadership model prioritized both structure and culture. According to a feature in *The Nation*, his reform not only digitized FMC’s operations and expanded service capacity but also created a ripple effect adopted by over 70 other federal institutions [1]. This is a testament to how localized planning, when done with clarity and conviction, can influence national health trajectories.

*A* *Practical* *Blueprint* *for* *Emerging* *Leaders*

For the next generation of clinicians hoping to improve specialty services, whether in a surgical, medical, or allied specialties, Dr. Dada’s advice is both timeless and urgent: don’t just work in the system but scan it; don’t just recognize problems; rather, structure them; and don’t just push change; instead, plan for it, measure, and scale it.

This is more than a leadership philosophy. It is a grounded, replicable health approach that belongs at the center of every public health curriculum and LMIC health system strategy.

“In every stage of life,” he reminds us, “you need to plan.”

*Legacy* *Through* *Process,* *Not* *Personality*

Dr. Dada’s success, by his admission, was not the result of bold speeches or dramatic breakthroughs. It was the result of a patient and structured implementation. His quiet refusal to improvise institutional direction has become his most enduring signature, a model of leadership that centers systems over spotlight and structure over speculation.

In an era when many health leaders face pressure to act quickly or symbolically, his message is both rare and refreshing. A lasting impact requires a plan.

Conclusion

This interview-derived leadership narrative demonstrates how a specialty clinician operationalized planning accountability and digital modernization to drive hospital-wide quality improvement in a resource-constrained setting. FMC Ebute-Metta’s transformation shows that internal leadership can reduce dependence on donor-driven initiatives when structure, trust, and governance align. While these insights are drawn from a single-case perspective, based primarily on a consented interview and secondary institutional reports, and may not be fully generalizable, they highlight strategies for clinician-led leadership that could inform policy and institutional change in similar low-resource settings. Future scale across LMIC hospitals will require financing reform, interoperable health data, and leadership models that integrate clinical insight with strategic management.

## References

[1] (2025). The Nation. Adedamola Dada bows out as CMD of Federal Medical Centre Ebute Metta in blaze of unparalleled adulation. https://thenationonlineng.net/adedamola-dada-bows-out-as-cmd-of-federal-medical-centre-ebute-metta-in-blaze-of-unparalleled-adulation/.

[2] (2025). Nigeria Health Watch. FMC Ebute Metta is restoring Nigeria’s confidence in public health centres. https://articles.nigeriahealthwatch.com/fmc-ebute-metta-is-restoring-nigerias-confidence-in-public-health-centres/?amp=1.

[3] Federal Medical Centre, Ebute-Metta Ebute-Metta (2025). The Rebirth of Federal Medical Centre Ebute-Metta, Lagos: 2017-2025.

[4] (2025). The Guardian. Expert harps on collaboration, teamwork for improved patient care. https://guardian.ng/features/health/expert-harps-on-collaboration-teamwork-for-improved-patient-care/.

[5] (2025). The Guardian. Patients commend e-medical registration process at FMC. https://guardian.ng/news/patients-commend-e-medical-registration-process-at-fmc/.

[6] (2025). Business Day. Ademola Dada: The surgeon driving rare efficiency in Nigeria’s public health. https://businessday.ng/health/article/ademola-dada-the-surgeon-driving-rare-efficiency-in-nigerias-public-health/.

[7] (2025). Vanguard. FMC Ebute Metta: We solve problems, not just manage them - Dr Adedamola Dada. https://www.vanguardngr.com/2025/05/fmc-ebute-metta-we-solve-problems-not-just-manage-them-dr-adedamola-dada/.

[8] World Health Organization (2010). The World Health Report: Health Systems Financing: The Path to Universal Coverage.

[9] (2025). Dada highlights milestones as FMC Medical Director. https://sundiatapost.com/dada-highlights-milestones-as-fmc-medical-director/.

[10] (2025). 30,000 patients seek care in FMC Ebute-Metta daily, MD reveals. https://healthwise.punchng.com/30000-patients-seek-care-in-fmc-ebute-metta-daily-md-reveals/.

